# An evaluation of the efficacy of a topical gel with Triester Glycerol Oxide (TGO) in the treatment of minor recurrent aphthous stomatitis in a Turkish cohort: A randomized, double-blind, placebo-controlled clinical trial

**DOI:** 10.4317/medoral.21469

**Published:** 2017-02-04

**Authors:** Duygu Ofluoglu, Sertan Ergun, Saman Warnakulasuriya, Filiz Namdar-Pekiner, Hakkı Tanyeri

**Affiliations:** 1 DDS, PhD. Istanbul University, Faculty of Dentistry, Department of Oral and Maxillofacial Surgery; 2 DDS, PhD, Assoc. Prof. Istanbul University, Faculty of Dentistry, Department of Oral and Maxillofacial Surgery; 3 Prof Dr. King’s College London, Department of Oral and Maxillofacial Surgery; 4 PhD, Prof Dr. Marmara University, Faculty of Dentistry, Department of Oral and Maxillofacial Radiology; 5 Prof Dr. Istanbul University, Faculty of Dentistry, Department of Oral and Maxillofacial Surgery

## Abstract

**Background:**

Triester glycerol oxide gel (Protefix® Queisser Pharma, Germany) is a new topical agent that has the property of adherence to the oral mucosa by forming a lipid film which protects against mechanical trauma and may help to reduce oral tissue moisture loss and inflammation. The aim of this clinical trial was to determine the efficacy of a topical TGO gel and to also compare it with triamcinolone acetonide pomade in the treatment of minor recurrent aphthous stomatitis.

**Material and Methods:**

This study was a randomized, double-blind, placebo-controlled clinical trial and 180 patients with the complaint of minor aphthous ulcers were enrolled in this study. The sociodemographic data and clinical characteristics of the ulcer were collected by questionnaire. Ulcer size and pain level measurements were performed and the efficacy indices for ulcer pain and size were calculated at day 0,2,4,6 by the same investigator.

**Results:**

Significant differences were not detected among the demographics and ulcer histories including age, gender, onset of ulcer, mean healing time, family RAS history and ulcer localization between three groups. The pain score in TGO group was found statistically lower at day 2,4, and 6. Efficacy index and improvement rate of TGO group, regarding pain score, was higher than the other two groups at day 2 and 4. The reduction in ulcer size was statistically higher in TGO group than the other two groups at day 4 and 6.

**Conclusions:**

Topical application of TGO gel could decrease pain intensity, accelerate ulcer healing without any side effects, utilizing an easy appliable and accessible procedure. Therefore TGO gel could be a well-tolerated, safe, topical therapeutic agent in the clinical practice of RAS treatment.

**Key words:**Topical therapy, triester glycerol oxide, triamcinolone acetonide, minor recurrent aphthous stomatitis.

## Introduction

Recurrent aphthous stomatitis (RAS) is a multifactorial chronic inflammatory disorder, characterized by recurrent, round or ovoid, painful ulcerations of the non-keratinized mucosa with a shallow necrotic center covered by a pseudomembrane and surrounded by an erythematous halo. RAS is divided into three varieties; minor, major and herpetiform of which the most common form is the minor variety (1-3). Although the episodes are generally transitory and self-limited, the symptoms can be disturbing, incapacitating and can affect patient’s quality of life (4). The prevalence of RAS is between 5% and 25% in the general population, but can be as high as 50% to 60% in different ethnic or socioeconomic groups (1,5).

Although several local and systemic causative factors including stress, local trauma, cessation of smoking, allergic agents, genetic background, infections, hematinic deficiencies, immune dysregulation, nutritional deficiencies, hormonal disturbances have been proposed its etiology remains unknown (6). RAS has been histologically characterized by varying degrees of neutrophils and mononuclear cell infiltration in the lamina propria and the inflammatory process plays an important role in RAS. The pain that is experienced might derive from the excessive inflammation and physiochemical irritation of afferent nerve endings at the junction of the epithelial and subepithelial layers (1,4). Because the definitive etiology and pathogenesis is not entirely understood, there is no specific and reliable therapy and the management of RAS still poses a complicated problem for both clinicians and patients worldwide.

The current therapeutic approaches aim to relieve pain, alleviate inflammation, decrease functional disability, promote ulcer healing as well as the reduction of the ulcer duration, frequency of recurrences and an increase in disease-free period. The best treatment is that which will control the ulcers for the longest time with minimum side effects. The treatment modality should be determined by disease severity, patient’s medical history, the frequency of flare-ups, size and number of ulcers and the patient’s ability to tolerate the medication. Several topical medicaments including corticosteroids, antibiotics, local analgesics, astringents, and laser therapy have been used for treatment (7,8). Systemic medications such as colchicine, levamisole, dapsone, thalidomide, pentoxifylline have been tried if topical therapy is ineffective. Although systemic medications are more effective, several side effects limit their long-term and extensive usage, therefore topical agents are still the first choice of treatment (8,9).

Triamcinolone acetonide (TA) a medium to high potency corticosteroid, is a fluorinated prednisolone derivative, considered an intermediate-acting glucocorticoid and available as a cream (0.1%) and as an ointment (0.1%) for topical use in medical practice. The absorption rate varies from 1% to 36% in different parts of the body and increases via damaged, inflammed or dressed skin. Metabolism of triamcinolone after topical application is dermal. The small amount which may enter systemic circulation is metabolized in the liver and when used in oral cavity, rarely there is a risk to lead adverse reactions such as candidiasis and/or atrophy of the oral mucosa (2,10). Topical TA ointment was shown to be effective in the treatment of aphthous lesions in the literature (10,11). Triamcinolone acetonide pomade, *(Kenacort-A Orabase® Pomade, 0,1% Triamsinolon acetonide, Bristol-Myers Squibb Ilacları Inc. Istanbul, Turkey)* is a slow release glucocorticoid pomade that forms a protective layer over the ulceration and exerts an anti-inflammatory action and has been used widely in aphthous ulcer treatment in Turkey for many years. Although new different topical agents were launched in recent years and alternative topical medicines with decreased side effects are gaining more attention, TA pomade is still the most commonly prescribed and best known agent in aphthous ulcer treatment for both the clinicians and the patients in Turkey.

Triester glycerol oxide (TGO) gel (Protefix® Queisser Pharma, Germany) is a topical agent that has the property of adherence to the oral mucosa by forming a lipid film which protects against mechanical trauma and may help to reduce oral tissue moisture loss and inflammation (12). Following its use a significant reduction of the number of erythrocytes and inflammatory cells and a significant increase of mature epithelial cells of oral mucosa were reported (13). TGO gel also has been shown to reduce RAS related symptoms, such as pain and was found effective in lengthening the duration of recurrence time (13). The objective of this randomized, double-blind, placebo-controlled clinical trial was to determine the efficacy of a topical TGO gel and also compare with TA pomade in the treatment of minor recurrent aphthous stomatitis in a group of Turkish patients.

## Material and Methods

- Materials and Blinding

TA pomade *(Kenacort-A Orabase® Bristol-Myers Squibb Ilacları Inc. Istanbul, Turkey)* contains 0.1% triamcinolon acetonide as an active ingredient and gelatin, pectin, carboxymethylcellulose sodium in plasticized hydrocarbon gel, a polyethylene and mineral oil gel base as an inactive substances. TGO gel (Protefix® Queisser Pharma, Germany) contains 92.67% triester glycerol oxide, where as placebo gel contains 92.67% corn oil and both gels contain 7% silica, colloid anhydrous, 0.2% clove oil, 0.1% saccharin sodium and 0.01% peppermint oil. All three agents were packed in the same sized, white colored soft tubes of 5g each. A single investigator (F.P) who was blind to the study protocol and subjects, randomly numbered these tubes and medications. Then, the numbers of the tubes were assigned to patients’ numbers by using a computer-generated number list and the tubes were then delivered to the clinical investigators who were blind to the treatment agents.

- Subjects and study design

This study was a randomized, double-blind, placebo-controlled clinical trial, approved by the Ethical Committee of Marmara University Faculty of Medicine (Project No: 09.2016.116). Based on the inclusion and exclusion criteria, 180 patients who were referred to the Istanbul University, Faculty of Dentistry, Department of Oral and Maxillofacial Surgery with the complaint of aphthous ulcers were enrolled in this study and all of the participiants received written and verbal information about the study and signed a detailed informed-consent form voluntarily. Randomly allocated subjects received one of these three agents; TA pomade, TGO gel or placebo gel.

The diagnosis of RAS was based on the anamnesis and clinical examination. Participants fullfilled the following inclusion criteria; being willing to participate in the study and signed the informed consent form; being between the ages of 18 and 65; having a history of RAS for at least two years with a frequency of at least one outbreak every two months; having only one well-demarcated ulcer in an easily accessible area of the mouth for less than 48 hours’ duration and reporting pain sensation without any anesthesia or paresthesia.

The exclusion criteria were as follows; pregnancy and lactation; having a hematological deficiency such as anemia, iron, vitamin B12 and/or folic acid deficiency that could pose a risk for RAS; systemic diseases such as ulcerative colitis, Crohn’s disease, Behçet’s syndrome in which RAS is part of their clinical presentation; alcohol and smoking consumption; allergy history; treatment of ulcers with systemic steroids, vitamins, antibiotics, antihistamines, oral retinoids or immunomodulatory agents within three months before study entry; and use of nonsteroidal anti-inflammatory drugs or mouthwash for ulcer treatment prior to 72 hours of study entry.

The sociodemographic data and clinical characteristics of the ulcer were collected by questionnaire regarding age, gender, the mean disease duration (onset of ulcer), the mean healing time of previous ulcers, genetic background including family RAS history, and the localization of the current ulcer. Ulcer size was calculated as follows; the distance between two opposite outside edges of the white border was measured by using a calibrated periodontal probe with milimeter markings. Two measurements approximately 90 degrees from each other were obtained and then multiplied to present the cross-sectional area of the ulcer. To evaluate pain level, a visual analog scale (VAS) consisting of a 10-cm horizontal line between the poles of “no pain (0)” to “unbearable pain (10)” was used. Patients were requested to mark the line with a vertical mark at the point that best represented the present pain level of the ulcer (14). Ulcer size and pain level measurements were recorded by the same investigator (D.O) in all patients at each appointment (Day 0,2,4,6).

The efficacy indices (EI) for ulcer pain and size were calculated using the following formula; Vx referring to values measured at determined days (in this study, at days 2,4 and 6) by the investigator and V1 referring to the baseline value measured at day 0.

EI=[(Vx-V1) ÷V1]×100% (4,15).

Efficacy indices (EI) were evaluated on a 4-rank scale:1)healed; EI ≥95%, 2)marked improvement; EI ≥70% to <95%, 3)moderate improvement; EI ≥30% to <70%, 4)no improvement; EI <30%.

Evaluation of marked improvement rate (MIR) referred to (1)+(2) (EI ≥ 70%) while evaluation of improvement rate (IR) referred to (1)+(2)+(3) (EI ≥ 30%) (14).

After questionnaire form was filled out and clinical examination was carried out, the ulcer size and pain were measured by the same investigator (D.O) in all patients before the first application of the agents (day 0). Then another investigator (S.E) who was blind to the group and agent assignment, applied the chosen agent to the ulcer to show the correct application of the agent. All patients were instructed to rinse their mouth with tap water prior to the administration of the agent and apply the agent to the ulcer 4 times per day (after meals and before bed time) for 7 days (day 0 to day 6) and it was recommended not to eat or drink anything for 30 minutes after application of the agent in all groups. The participants were requested to visit our clinic on the morning (9-11 am) of days 2,4 and 6 for ulcer size and pain evaluation and strictly warned not to use any other products for the treatment of aphthous ulcers while participating in this study. At the end of therapy, all patients were also asked to report any adverse effects of the agents.

- Statistical analyses

The sample size was determined using G*Power 3.0.10 software. A total of 29 ulcer per group were chosen considering Power: 0.80, a:0.05, effect size: 0.339 and SD: 2 for pain score. All of the data was analyzed with IBM SPSS Statistics 22.0 software program and significance was evaluated at a level of *p*<0.05. During the assessment of the study data, conformity of the parameters to the normal distribution was assessed by the Shapiro Wilks test. During the evaluation of the study data, regarding the comparisons of quantitative data as well as descriptive statistical methods (Mean, Standard deviation), One-way Anova test was used for the intergroup comparisons of parameters with normal distribution and Tukey HDS test was used for the determination of the group causing difference. Kruskal Wallis test was used for the intergroup comparisons of parameters without normal distribution and Mann Whitney U test was used for the determination of the group causing difference. Paired Samples t test was used for the in-group comparisons of parameters with normal distribution. Wilcoxon Signed Ranks test was used for the in-group comparisons of parameters without normal distribution. Chi-Square test was used for comparison of qualitative data.

## Results

A total of 180 patients were enrolled in the study and 20 patients dropped out due to violations of the study protocol therefore 53 subjects in TA group, 56 subjects in TGO group and 51 subjects in placebo group completed the study (total 160). We evaluated demographic and effectiveness datas excluding these subjects. Statistically significant differences were not detected among the demographics and ulcer histories including age, gender, onset of ulcer, mean healing time, family RAS history and ulcer localization between three groups (*p*>0.05). 51.3% (n=82) were females and 48.8% (n=78) were males. The age of participants ranged between 18-65 and the mean age was 38.76±13.03 years.

- Moderation of pain

**Figure 1:**
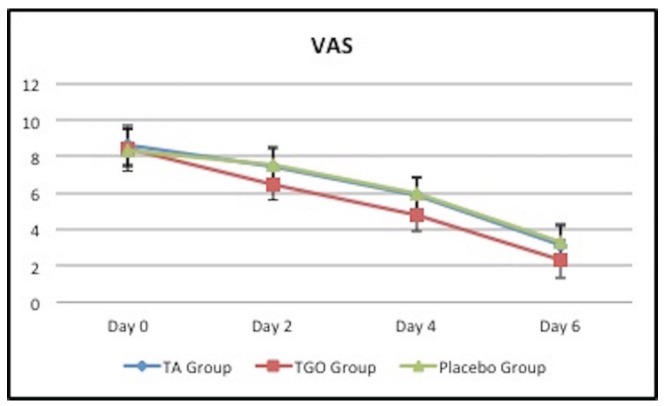
Comparison of mean ulcer pain scores among the three groups.

Although the mean ulcer pain scores of three groups matched well at study entry (*p*>0.05), significant group differences were found at the later visits (day 2,4,6) (*p*<0.01). The ulcer pain scores (VAS) of three groups decreased with time however, the pain score in TGO group was statistically lower than that of the TA and placebo groups at days 2,4 and 6 (*p*<0.01), there was no significant difference between TA and placebo groups (*p*>0.05) ( Table 1, Fig. 1).

**Table 1:**
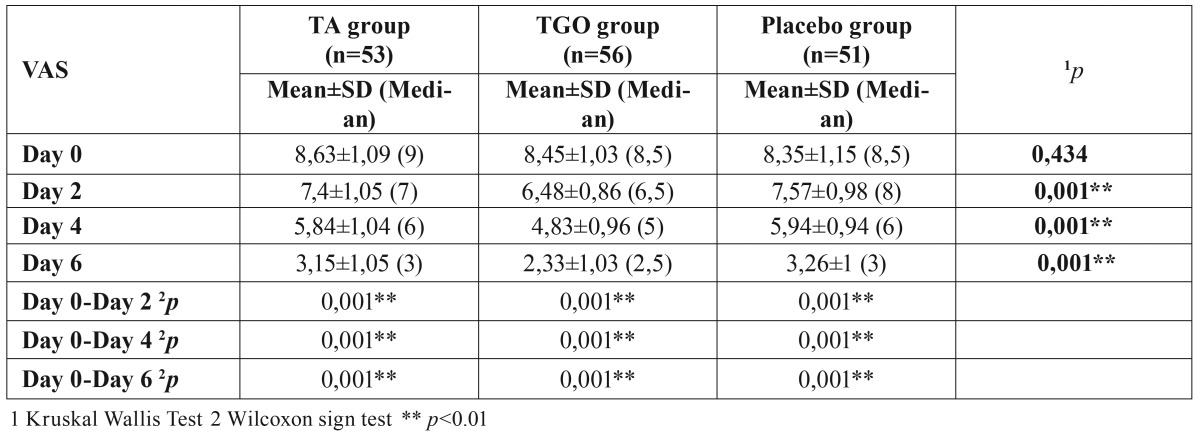
Comparison of mean ulcer pain scores among the three groups.

On the second day, the efficacy index of the TGO group was found much greater than that of TA and placebo groups (*p*<0.01). Also there was a significant difference between TA group and placebo group (*p*:0.001; *p*<0.01). The TGO group had a significantly higher “improvement rate” (10.7% vs 0%,*p*<0.003) when compared with TA and placebo groups whereas there was no significant difference between TA and placebo groups (*p*>0.05) ( Table 2).

**Table 2:**
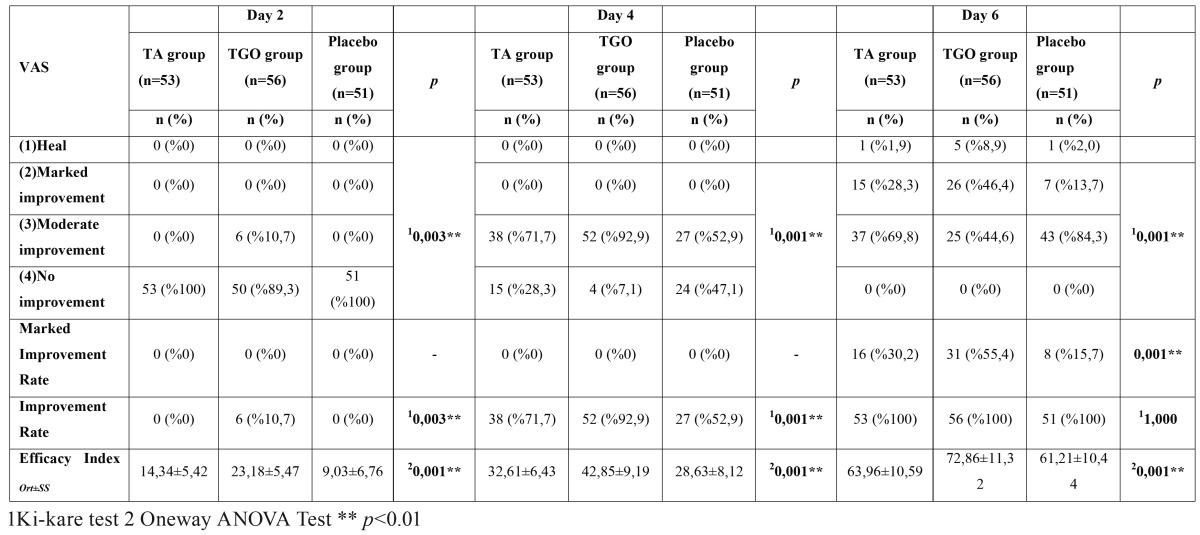
Efficacy of VAS reduction.

On the fourth day, the efficacy index of the TGO group was found much greater than that of TA and placebo groups (*p*<0.01) and had a significantly higher “improvement rate” (92.9% vs 71.7% and 52.9%%, *p*<0.001) when compared with TA and placebo groups. The efficacy index and “improvement rate” of TA group was significantly higher than that of placebo group (*p*<0.05) ( Table 2).

On the sixth day, the number of patients whom totally healed (8.9% vs 1.9% and 2%) and showed marked improvement (46.4% vs 28.3% and 13.7%) in TGO group were statistically higher than the TA and placebo groups (*p*<0.05; *p*<0.01) whereas no statistically significant difference was found between TA and placebo groups (*p*>0.05). TGO group sustained a significantly higher efficacy index (*p*<0.01) but there was no significant difference between TA and placebo groups in terms of efficacy index (*p*>0.05). The “improvement rate” was similar in all groups but “marked improvement rate” of TGO group was found statistically higher than that of TA and placebo groups (*p*<0.01) ( Table 2).

- Reduction in ulcer size

**Figure 2:**
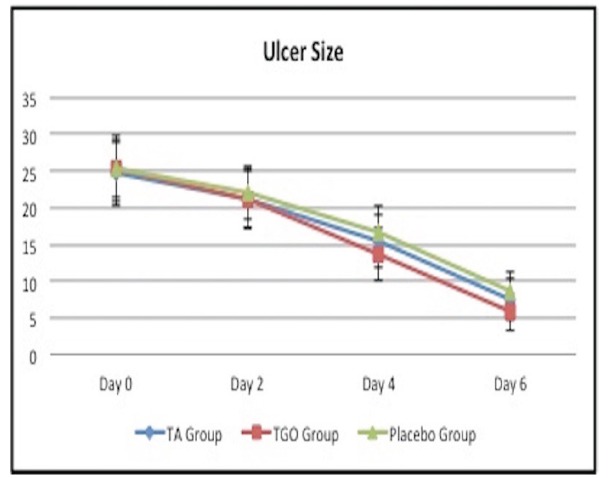
Comparison of ulcer size among the three groups.

There was no significant difference among mean ulcer sizes of three groups at study entry and on the second day (*p*>0.05); but significant group differences appeared at day 4 and 6 (*p*<0.01). The mean ulcer size in TGO group was statistically lower than that of the TA and placebo groups at day 4 and 6 (*p*<0.01), while there was no significant difference between TA and placebo groups (*p*>0.05) ( Table 3, Fig. 2).

**Table 3:**
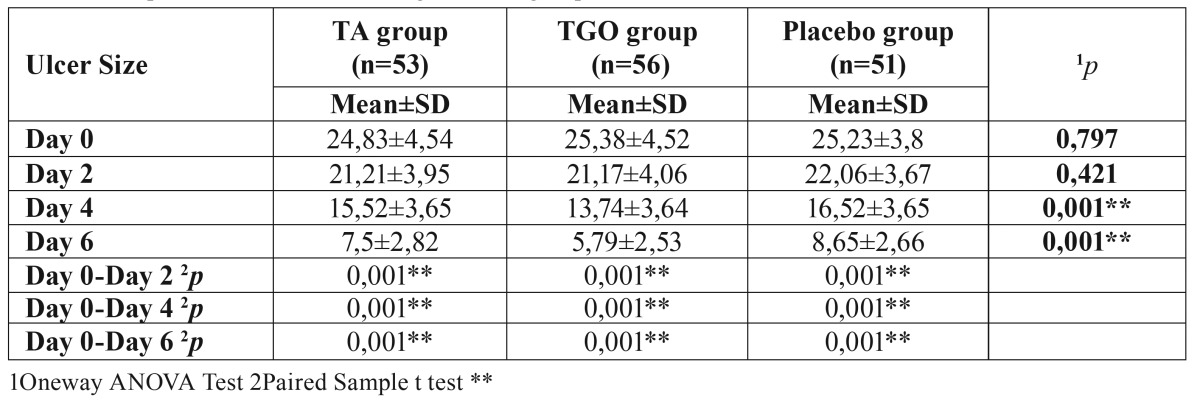
Comparison of ulcer size among the three groups.

On the second day, the efficacy index of the TGO group was found much greater than placebo group (*p*:0.001; *p*<0.01) but there was no significant difference between other groups (*p*>0.05). Although the “improvement rate” was similar in all groups (*p*>0.05) ( Table 4).

**Table 4:**
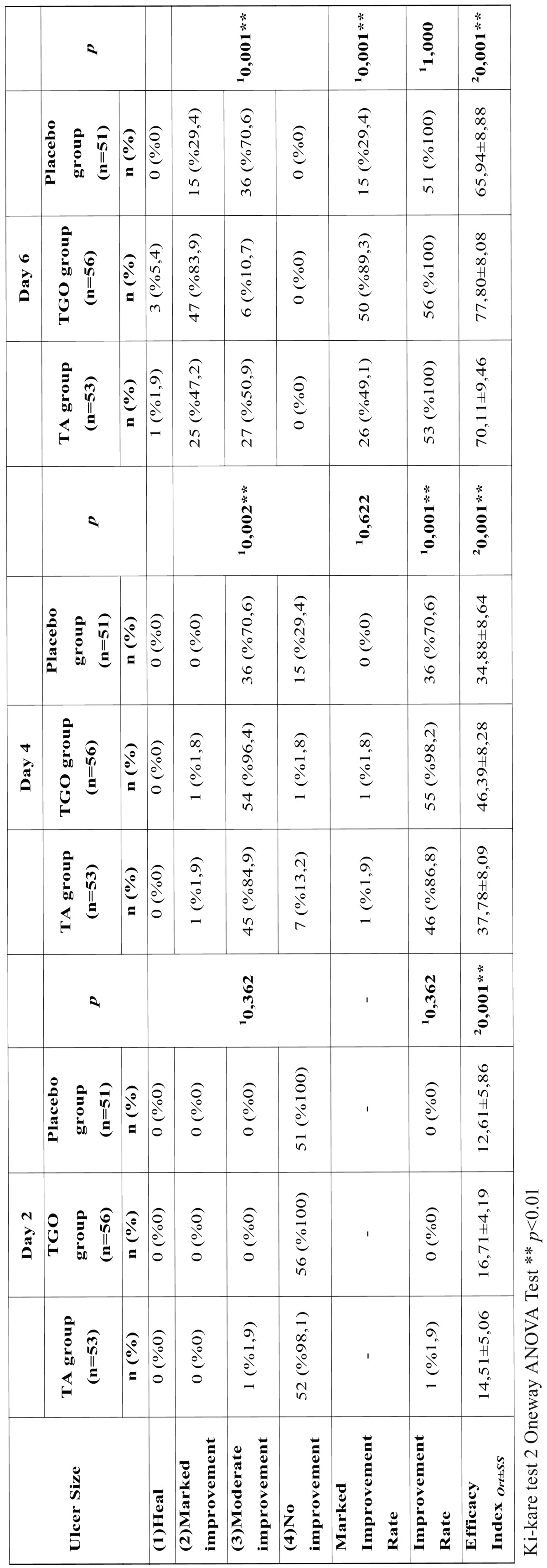
Efficacy of ulcer size reduction

On the fourth day, the efficacy index of the TGO group was found much greater than that of other groups (*p*<0.01) and had a significantly higher “improvement rate” (98.2% vs 86.8% and 70.6%, *p*<0.001) when compared with TA and placebo groups. There were no significant differences between TA and placebo groups in terms of efficacy index and “improvement rate” (*p*>0.05) ( Table 4).

On the sixth day, compared with TA and placebo groups, the TGO group maintained a significantly higher efficacy index (*p*<0.01) whereas a significant difference was found between TA and placebo groups (*p*:0.045;*p*<0.05) ( Table 4). Although the “improvement rate” was similar in all groups (*p*>0.05) “marked improvement rate” of TGO group was found statistically higher than that of other groups (*p*<0.01) and there was no difference between TA and placebo groups (*p*>0.05) ( Table 4).

All patients tolerated the agents and no side effect were reported during the study. Only 5 patients in TA group (9.43%) mentioned some difficulties in the application of the agent.

## Discussion

RAS is a complicated condition and the precise etiology still remains unknown. High frequency, worldwide distribution, wide age range, recurrent character of the disease and decreased quality of life of RAS patients have lead to a great deal of research into the etiology and treatment of this condition. Various treatment modalities, topical and systemic agents have been evaluated in different countries for many years (9). Although the exact mechanism of TGO in the treatment of RAS remains to be clarified, adherence to the oral mucosa by forming a lipid film protects against mechanical trauma and helps to reduce inflammation. The results of this study showed that TGO gel is benefical in pain relief, ulcer size reduction and the promotion of healing without any systemic or local side effects.

As known the first line treatment of RAS should always start with topical medications such as oral rinses, gels, patches, tablets or adhesive films which provide antiseptic, anti-inflammatory and analgesic effects (2,3,16,17). The most important agents in RAS treatment are topical corticosteroids. TA dental paste is a popular agent and is being widely used in the treatment of mild to severe RAS lesions at different concentrations ranging 0.05%-0.5% (the most effective concentration to be 0.1%) 3-10 times a day (2,18,19).

Several studies comparing the efficiency of different agents with TA were reported in the literature. Deshmukh and Bagewadi have compared the efficacy of curcumin, which is known for its strong antioxidant, antiseptic, antibacterial, anti-inflammatory, immunomodulatory and analgesic properties, and TA in the gel form in the treatment of RAS and they found similar reduction in size and number of ulcer in both groups (20). Bhalang *et al.*reported a higher effectiveness of 0.1% TA than acemannan, a polysaccharide extracted from Aloe vera, in the treatment of oral aphthous ulceration (21). Comparing TA 0.025% with chlorhexidine 0.12% applied topically, covered by a barrier of isobutyl cyanoacrylate, in the treatment of RAS, a controlled, randomized clinical study found a very significant difference in the reduction of the intensity and the perception of pain on different days when the two groups with medication were compared with the control group, but the difference was not significant when the two medicines were compared (18,22).

Hyaluronic acid (HA) gel is an alternative agent for topical treatment. The topical application of 0.2% HA gel seems to be an effective and safe therapy in patients with RAS (23). Different lasers were also used for RAS treatment, Tezel *et al.* (24) have suggested that the Nd:YAG laser has better patient acceptance, shorter treatment time, lower rates of pain and post-treatment adverse events among patients with RAS. Lalabonova *et al.* reported that to use low-level laser therapy for treatment of chronic RAS was better than those obtained in the group receiving pharmacotherapy. Pain and inflammation have been very effectively managed with LLLT and epithelization has been considerably accelerated (25).

Although several treatment modalities have been tried, RAS is one of the most common oral mucosal disease in Turkey and it still poses a complicated problem for both the clinicians and patients. In our study, as an alternative to TA pomade, a randomized, double-blind, placebo-controlled clinical trial has investigated the effectiveness of TGO gel (Protefix® Queisser Pharma, Germany) in the treatment of minor RAS. The results demonstrated that TGO gel not only resolved the pain of the patients but also reduced the ulcer size with no systemic or local side effects. Furthermore TGO gel was easy to use without any unfavorable taste and was easy for patients to apply. In this study, 10.7% of the patients who used TGO gel showed a moderate improvement in pain intensity within 2 days of the start of treatment, whereas in the TA and control group, none of the patients showed moderate improvement in the same period. The pain score in TGO group was statistically lower than that of the TA and placebo groups at day 2,4 and 6 whereas there was no significant difference between TA and placebo groups. Although the baseline ulcer size was similar in three groups at the beginning of the study, significant differences were detected after 4 days. The reduction in ulcer size of the TGO group was found much greater at day 4 and 6 when compared with TA and placebo groups. These results showed the efficacy of TGO gel in pain and ulcer size reduction.

Some curative effect for pain relief was also observed in placebo group which may be caused from the protective film layer produced by the placebo gel because it isolates the physical and chemical stimuli and promotes the healing process. Because all the patients were blind to the therapeutic agents, placebo may have also caused some psychologic effects. According to our knowledge this is the first randomized, double-blind, placebo-controlled clinical trial investigating the effects of TGO gel in the treatment of RAS. In a placebo controlled study, the efficacy of TGO gel in the treatment of the ulcerations related to new complete denture have been investigated and TGO gel found ineffective (13).

Given the inflammatory nature of RAS, our interpretation of these results is that the clinical beneficial effects of the TGO gel could be attributed to the ability to decrease inflammation and increase epithelization. Because we did not perform a cytological examination we could not know the exact role of TGO in the healing process of an ulcer. Further studies involving larger number sample sizes and evaluating the effect of TGO in a cellular base are recommended to clarify the mechanism and the efficacy of RAS on recurrence.

In conclusion, the present study findings demonstrate that topical application of TGO gel could decrease pain intensity, accelerate ulcer healing without any side effects and with an easily appliable and accessible procedure. Therefore TGO gel could be a well-tolerated, safe, topical therapeutic agent in clinical practice of RAS treatment.
